# Antiepileptogenesis after stroke—trials and tribulations: Methodological challenges and recruitment results of a Phase II study with eslicarbazepine acetate

**DOI:** 10.1002/epi4.12735

**Published:** 2023-06-12

**Authors:** Matthias J. Koepp, Eugen Trinka, Yee‐Haur Mah, Carla Bentes, Susanne Knake, Gian Luigi Gigli, José M. Serratosa, Johan Zelano, Luís M. Magalhães, Ana Pereira, Joana Moreira, Patrício Soares‐da‐Silva

**Affiliations:** ^1^ UCL Queen Square Institute of Neurology London UK; ^2^ National Hospital for Neurology and Neurosurgery London UK; ^3^ Department of Neurology Christian‐Doppler University Hospital, Paracelsus Medical University, Centre for Cognitive Neuroscience, Member of EpiCARE Salzburg Austria; ^4^ Neuroscience Institute, Christian‐Doppler University Hospital Paracelsus Medical University, Centre for Cognitive Neuroscience Salzburg Austria; ^5^ Institute of Public Health, Medical Decision‐Making and HTA UMIT – Private University for Health Sciences Medical Informatics and Technology Hall in Tyrol Austria; ^6^ King's College Hospital NHS Foundation Trust London UK; ^7^ School of Biomedical Engineering and Imaging Sciences King's College London London UK; ^8^ Reference Centre for Refractory Epilepsies (Member of EpiCARE) Hospital de Santa Maria‐CHULN Lisbon Portugal; ^9^ Department of Neuroscience and Mental Health (Neurology) Hospital de Santa Maria‐CHULN Lisbon Portugal; ^10^ Centro de Estudos Egas Moniz Faculdade de Medicina da Universidade de Lisboa Lisbon Portugal; ^11^ Department of Neurology, Epilepsy Centre Hessen Philipps‐University Marburg Marburg Germany; ^12^ Clinical Neurology Unit, Department of Medicine (DAME) University of Udine Udine Italy; ^13^ Department of Neurology and Laboratory of Neurology, Fundación Instituto de Investigación Sanitaria‐Fundación Jiménez Díaz Autónoma University Madrid Spain; ^14^ Centro de Investigacion Biomedica en Red de Enfermedades Raras (CIBERER) Madrid Spain; ^15^ Institute of Neuroscience and Physiology, Sahlgrenska Academy University of Gothenburg Gothenburg Sweden; ^16^ Wallenberg Centre for Molecular and Translational Medicine University of Gothenburg Gothenburg Sweden; ^17^ Department of Neurology Sahlgrenska University Hospital Gothenburg Sweden; ^18^ Bial—Portela & Cª, S.A. Coronado Portugal; ^19^ Department of Biomedicine Pharmacology and Therapeutics Unit, Faculty of Medicine University Porto Porto Portugal; ^20^ MedInUP—Center for Drug Discovery and Innovative Medicines University Porto Porto Portugal

**Keywords:** acute intracerebral hemorrhage or infarct, antiseizure medication, epilepsy, epileptogenesis, unprovoked seizure

## Abstract

There is currently no evidence to support the use of antiseizure medications to prevent unprovoked seizures following stroke. Experimental animal models suggested a potential antiepileptogenic effect for eslicarbazepine acetate (ESL), and a Phase II, multicenter, randomized, double‐blind, placebo‐controlled study was designed to test this hypothesis and assess whether ESL treatment for 1 month can prevent unprovoked seizures following stroke. We outline the design and status of this antiepileptogenesis study, and discuss the challenges encountered in its execution to date. Patients at high risk of developing unprovoked seizures after acute intracerebral hemorrhage or acute ischemic stroke were randomized to receive ESL 800 mg/d or placebo, initiated within 120 hours after primary stroke occurrence. Treatment continued until Day 30, then tapered off. Patients could receive all necessary therapies for stroke treatment according to clinical practice guidelines and standard of care, and are being followed up for 18 months. The primary efficacy endpoint is the occurrence of a first unprovoked seizure within 6 months after randomization (“failure rate”). Secondary efficacy assessments include the occurrence of a first unprovoked seizure during 12 months after randomization and during the entire study; functional outcomes (Barthel Index original 10‐item version; National Institutes of Health Stroke Scale); post‐stroke depression (Patient Health Questionnaire‐9; PHQ‐9); and overall survival. Safety assessments include the evaluation of treatment‐emergent adverse events; laboratory parameters; vital signs; electrocardiogram; suicidal ideation and behavior (PHQ‐9 question 9). The protocol aimed to randomize approximately 200 patients (1:1), recruited from 21 sites in seven European countries and Israel. Despite the challenges encountered, particularly during the COVID‐19 pandemic, the study progressed and included a remarkable number of patients, with 129 screened and 125 randomized. Recruitment was stopped after 30 months, the first patient entered in May 2019, and the study is ongoing and following up on patients according to the Clinical Trial Protocol.

**Key points**

- Animal models have demonstrated a potential antiepileptogenic effect for eslicarbazepine acetate (ESL).
- A Phase II study has been designed to assess whether ESL treatment for 1 month can prevent unprovoked seizures following stroke.
- Patients at high risk of developing post‐stroke epilepsy received ESL 800 mg/d or placebo, within 120 hours after stroke occurrence.
- Despite design and recruitment challenges, 125 patients have been randomized and the study is currently ongoing.
- The study will help address the current lack of evidence for the use of antiseizure medications to prevent post‐stroke epilepsy.

## 1 INTRODUCTION

Epilepsy affects at least 50 million people globally and is associated with significant morbidity and mortality.^1^, ^2^, ^3^ Available drug therapies suppress seizures, targeting the symptoms rather than causes of epilepsy, and none modifies the course or prevents the onset of the disease.^1^, ^2^, ^3^, ^4^ Although many patients achieve seizure freedom with existing antiseizure medications (ASMs), most require long‐term (often lifelong) treatment, adding to the serious impact of chronic epilepsy on their physical and psychosocial wellbeing, and also resulting in a substantial burden on the healthcare system.^2^, ^5^, ^6^, ^7^, ^8^ There is a pressing need for new therapies that can prevent the development of chronic epilepsy or modify its course after it presents.^2^, ^9^, ^10^

Stroke accounts for up to 11% of all epilepsy cases and is the leading cause in the elderly population, responsible for approximately 50% of newly diagnosed epilepsy cases in those aged >60 years.^11^, ^12^, ^13^ Following a stroke, 5%–15% of patients experience a seizure within 2 years,^12^, ^14^, ^15^ the risk of post‐stroke seizures increasing with the severity of the stroke.^16^ Post‐stroke seizures are classified as “early” (or acute symptomatic) if they occur within 7 days of the stroke, and as “late” (or unprovoked) if they occur later than this.^12^, ^17^, ^18^ Early seizures are likely to result from physiological or metabolic alterations caused by the stroke itself, whereas unprovoked seizures can occur weeks or years after the index event and are thought to result from epileptogenesis, which encompasses the process of how a previously non‐epileptic brain becomes functionally altered and biased towards the generation of abnormal paroxysmal electrical activity.^1^, ^12^ Occurrence of a single unprovoked seizure following a brain insult, such as stroke, is associated with an increased risk of seizure recurrence that is comparable to that following two unprovoked seizures, and, for this reason, the International League Against Epilepsy (ILAE) defines the occurrence of a single unprovoked seizure following a stroke as epilepsy.^19^ Post‐stroke epilepsy can cause significant morbidity; in addition to seizure‐related risks, the use of enzyme‐inducing ASMs is still common,^20^ which in turn detrimentally interacts with secondary stroke prophylaxis^21^ and has a high risk for side‐effects.^22^, ^23^ Post‐stroke epilepsy is also associated with reduced quality of life and survival.^24^, ^25^, ^26^

Evidence for the use of ASMs in the primary and secondary prevention of post‐stroke epilepsy is currently limited. A recent Cochrane review identified two randomized double‐blind studies with a total of 856 patients, concluding that ASMs were not shown to be effective in the primary prophylaxis of post‐stroke seizures.^27^ A third study was stopped after only 16 patients were recruited, with the authors concluding that it is not feasible to conduct a prophylactic study assessing the antiepileptogenic efficacy of a short ASM treatment period to prevent post‐stroke epilepsy.^28^ There are currently no approved therapies for individuals at risk of epileptogenesis,^2^, ^29^ and insufficient evidence to support the use of ASMs for the prevention of late, unprovoked seizures following stroke.^4^, ^27^, ^28^

Eslicarbazepine acetate (ESL) is a once‐daily ASM that is approved in Europe and the USA for the treatment of focal‐onset seizures as monotherapy or adjunctive therapy.^30^, ^31^ In preclinical models, ESL suggested antiepileptogenic effects, mediated by effective inhibition of high‐ and low‐affinity hCaV3.2 inward currents.^32^, ^33^ For example, in a murine pilocarpine model of chronic epilepsy, transitory ESL treatment was shown to significantly reduce the frequency and duration of epileptiform discharges during the chronic stage, and ESL treatment was additionally shown to attenuate neuronal loss, significantly lessening coordination impairment.^32^

Here we present an overview of an ongoing Phase II, multicenter, randomized, double‐blind, placebo‐controlled study, designed to assess whether treatment with ESL for 1 month, starting within 120 hours after stroke occurrence, can prevent unprovoked seizures, compared to placebo treatment. We also discuss the challenges encountered in conducting antiepileptogenesis studies, particularly in patients at high risk of developing post‐stroke epilepsy.

## 2 MATERIALS AND METHODS

### 2.1 Study design

**FIGURE 1:**
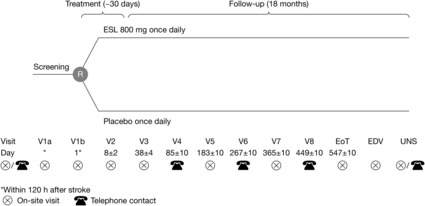
Study design. EDV, early discontinuation visit; EoT, end of trial visit; ESL, eslicarbazepine acetate; R, randomization; UNS, unscheduled visit; V, visit.

Study BIA‐2093‐213 is a Phase II, multicenter, double‐blind, randomized, placebo‐controlled, parallel‐group study in patients at high risk of developing unprovoked seizures after acute intracerebral hemorrhage or an acute ischemic stroke (Figure 1). Patients can receive therapies for stroke treatment according to local clinical practice at any time during the study. Following the initial screening (visit [V] 1a), eligible patients are randomized (1:1) to receive treatment with ESL 800 mg/d or placebo. In patients with moderate renal impairment at Visit 1a (defined as an estimated glomerular filtration rate [eGFR] of 30–60 mL/min/1.73 m^2^), ESL should be initiated at 400 mg/d and increased to 800 mg/d as soon as the eGFR has improved to >60 mL/min/1.73 m^2^. In patients developing moderate renal impairment while receiving the study drug, ESL dosing should be adjusted to 400 mg/d, if applicable. If renal function worsens to an eGFR <30 mL/min/1.73 m^2^, the study drug should be discontinued (independent of dose) without down‐titration. Treatment is initiated (V1b) within 120 hours after the primary stroke occurrence, or since last time seen well. Treatment continues until Day 30 after randomization and is then tapered off. Patients are followed up until 18 months after randomization. Patients can concomitantly receive antiepileptic therapies, except commercially available ESL or oxcarbazepine, until Day 30. Concomitant antiepileptic therapies must be discontinued, and down‐titration must start according to the respective Summary of Product Characteristics. If antiepileptic therapies are not already discontinued before Day 30, down‐titration must commence on Day 31 at the latest. Further visits are performed 7 days (V2, on‐site), 37 days (V3, on‐site), 12 weeks (V4, telephone), 26 weeks (V5, on‐site), 38 weeks (V6, telephone), 52 weeks (V7, on‐site), 64 weeks (V8, telephone) and approximately 18 months (End of Trial visit, on‐site) after V1b. Patients who experience a first unprovoked seizure discontinue study treatment and are treated at the discretion of the investigator until 18 months after randomization, except with commercially available ESL. Patients who discontinue study participation prematurely are asked to attend the site for an early discontinuation visit, and unscheduled visits are performed at the discretion of the investigator, as necessary. Patients or their caregivers (as applicable) are instructed to contact the site immediately after a seizure has occurred and to document seizure information in a patient diary.

### 2.2 Ethics

The study is being conducted in accordance with the Declaration of Helsinki on Ethical Principles for Medical Research Involving Human Patients, adopted by the General Assembly of the World Medical Association, Fortaleza, Brazil 2013, as well as with the valid national/local laws of the participating countries, with the International Council for Harmonisation of Technical Requirements for Pharmaceuticals for Human Use Harmonised Tripartite Guideline for Good Clinical Practice (E6), and with the European Commission Directives 2001/20/EC and 2005/28/EC. The protocol was submitted to the responsible Independent Ethics Committees (IECs) and their written unconditional approval was obtained before the start of the study. IECs are informed of all subsequent protocol amendments that are likely to affect the safety of the patients or the conduct of the study, and of all suspected unexpected serious adverse reactions occurring during the study. The study is being conducted in 21 sites in seven European countries (Austria, Germany, Italy, Portugal, Spain, Sweden, and the United Kingdom) and Israel. The study is funded by Bial – Portela & Cª, S.A. and is registered on the European Union Drug Regulating Authorities Clinical Trials Database (EudraCT; number 2018‐002747).

### 2.3 Study population

The study population comprises adult stroke patients at high risk for an unprovoked seizure after acute intracerebral hemorrhage or acute ischemic stroke. See Table 1 for more details of key inclusion and exclusion criteria.

**TABLE 1:** Key inclusion and exclusion criteria.

Inclusion criteria	Exclusion criteria
Informed consent (from patient or patient's legal representative)	Contraindication to ESL^a^
Men and women aged ≥18 y	Known Han Chinese or Thai ancestry
Confirmation of one of the following, by MRI or CT: Acute ischemic stroke or intracerebral hemorrhagic stroke **with** An acute symptomatic seizure until 120 h post‐stroke **and** Cerebral cortex involvement *OR* Acute ischemic stroke **with** NIHSS ≥11 **and** Cerebral cortex involvement **and** Large‐artery atherosclerosis **and/or** territory of MCA *OR* Acute ischemic stroke **with** NIHSS 4–10 **and** Cerebral cortex involvement **and** Large‐artery atherosclerosis **and** Territory of MCA *OR* Acute intracerebral hemorrhagic stroke **with** Cerebral cortex involvement **and** Volume of intracerebral hemorrhage >10 mL	
	Use of oxcarbazepine or ESL (except in this trial)
	History of ASM use within previous 2 y
	History of unprovoked seizures prior to index stroke event
	History of previous clinical cerebral cortical stroke (other than index stroke event) within 2 y prior to V1a
	Sinus venous thrombosis
	Spontaneous subarachnoid hemorrhage (e.g., due to aneurysmatic or arteriovenous malformation)
	Impaired pre‐stroke level of function (i.e., mRS score >3 prior to first stroke occurrence)
Time of stroke occurrence known and V1b planned within 120 h since known time of stroke, or since last time seen well	Severe hepatic impairment
	eGFR <30 mL/min/1.73m^2^ at V1a
Brain scan analysis reliably excluded structural brain lesions mimicking stroke (e.g., cerebral tumor, brain abscess)	Known or suspected acute or chronic alcoholism, delirium tremens, or toxic psychosis
	History of suicidal ideation or suicide attempt within previous 3 y
*For women:* female patients of childbearing potential must not be pregnant, and sexually active females must use a medically acceptable, effective, non‐hormonal method of contraception up to the end of the current menstrual cycle after stopping treatment Female patients without childbearing potential are eligible	Presence of any other significant or progressive/unstable medical condition that would compromise trial participation, safety, or compliance (investigator's opinion)
	*For women:* pregnancy or breastfeeding

Abbreviations: ASM, antiseizure medication; CT, computed tomography; eGFR, estimated glomerular filtration rate; ESL, eslicarbazepine acetate; MCA, middle cerebral artery; MRI, magnetic resonance imaging; mRS, modified Rankin Scale; NIHSS, National Institutes of Health Stroke Scale; V, visit.

^a^ Known hypersensitivity to ingredients of ESL formulation or other carboxamide derivatives (e.g., oxcarbazepine and carbamazepine), or second or third atrioventricular block.

### 2.4 Study assessments

The primary efficacy endpoint is the proportion of patients experiencing a first unprovoked seizure within the first 6 months after randomization (“failure rate”). Deaths before month 6 after randomization or patients without evaluable assessment of the primary endpoint will be counted as treatment failures. Secondary efficacy assessments comprise: the proportion of patients who experience a first unprovoked seizure during the first 12 months after randomization (“12‐month failure rate”); the proportion of patients who experience a first unprovoked seizure during the course of the study (i.e., until 18 months after randomization; “18‐month failure rate”); the number of acute symptomatic seizures; time to first unprovoked seizure after randomization; time to first unprovoked seizure after stroke occurrence; the number and 4‐week rate of unprovoked seizures; functional outcomes, assessed using the Barthel Index original 10‐item version^34^ and the National Institutes of Health Stroke Scale (NIHSS)^35^; post‐stroke depression, assessed using the Patient Health Questionnaire‐9 (PHQ‐9)^36^; and overall survival.

Safety assessments include the evaluation of: treatment‐emergent adverse events, including findings from physical and neurological examinations; laboratory parameters (hematology, biochemistry, estimated glomerular filtration rate, coagulation, and urinalysis); vital signs; electrocardiogram; and suicidal ideation and behavior (assessed using question 9 of the PHQ‐9).^36^ Electroencephalogram (EEG) evaluation is included as an optional exploratory assessment.

### 2.5 Randomization and blinding

After eligibility is confirmed at V1b, patients are randomized (1:1) to receive ESL or placebo. The randomization list is produced using validated Statistical Analysis System (SAS) software (SAS Institute Inc.). ESL and placebo tablets are identical in size, color, taste, and appearance. The packaging and labeling do not allow for any distinction between test drug and placebo. No person involved in conducting the study may have access to the randomization code before the blind is officially broken. Unblinding will not occur unless an actual emergency occurs, and knowledge of the patient's randomization code affects his/her medical treatment. A record, signed by the investigator, will be made of the date, time, and reason for breaking the blind.

### 2.6 Statistical analysis

#### 2.6.1 Sample size calculation

Based on retrospective historical data,^16^, ^37^ 26% of patients are expected to experience a first unprovoked seizure (i.e., the failure rate) within the first 6 months following a stroke with the standard of care. As this is a pilot study, no empirical estimate of the treatment effect in patients randomized to ESL is available. The study is planned to have ≥80% power to demonstrate a significantly lower failure rate with ESL versus placebo under the following assumptions: an expected failure rate (including death, and other reasons for missing data before the first unprovoked seizure if a seizure occurred) of 26% under placebo within the first 6 months following a stroke; an expected failure rate (including death, and other reasons for missing data before the first unprovoked seizure if a seizure occurred) of 8% under ESL within the first 6 months following a stroke. Under these assumptions, 100 patients per treatment arm will ensure ≥80% power to demonstrate a significantly lower failure rate under ESL versus placebo (5% level of significance). Approximately 220 patients will be screened and approximately 200 will be randomized in the study.

#### 2.6.2 Analysis populations

The Enrolled Set is defined as all patients enrolled in the study. The Safety Set and Full Analysis Set (FAS) are both defined as all randomized patients treated with at least one dose of study drug. The Per‐Protocol Set is defined as all patients in the FAS without major protocol deviations. The EEG Analysis Subset is defined as all patients in the FAS with a baseline and post‐baseline (+78 weeks) EEG recording available. All efficacy analyses will be based on the FAS and Per‐Protocol Set, and all safety analyses will be based on the Safety Set. Demographic and baseline characteristics will be presented for the FAS, Per‐Protocol Set, and Safety Set. All listings will be presented for the Enrolled Set.

#### 2.6.3 Statistical tests

The primary efficacy endpoint (6‐month failure rate) will be assessed using a chi‐square test with continuity correction on the significance level of 5% (two‐sided). Twelve‐ and 18‐month failure rates will be assessed using the same approach as for the primary efficacy endpoint. Time to event (i.e., time to first unprovoked seizure) analyses will be assessed using Kaplan–Meier methodology. Cause‐specific cumulative incidence curves will evaluate the competing risk in case of death before the first unprovoked seizure. Descriptive statistics will be used to summarize the number of acute symptomatic seizures, the number and 4‐week rate of unprovoked seizures, and results for the Barthel Index, NIHSS, and PHQ‐9. Demographic and baseline characteristics, safety assessments and the exploratory endpoint will be summarized using descriptive statistics.

## 3 CURRENT STATUS

The study was initially planned to randomize approximately 200 patients. The first patient entered the study in May 2019. The recruitment was estimated to be completed in June 2020 but was prolonged several times and finally stopped after 30 months, at which point 129 patients had been screened and 125 randomized.

## 4 DISCUSSION

The development of preventative or disease‐modifying treatments for post‐stroke epilepsy is urgently needed in view of the high incidence of stroke, and the negative impact epilepsy has on recovery from stroke. Following stroke, epilepsy develops over days to years. This would allow treatment to prevent or modify post‐stroke epilepsy, but no such treatment exists. Only low‐level evidence is available to guide the clinical management of post‐stroke epilepsy, and current guidelines provide weak recommendations on prevention of post‐stroke seizures.^38^ Evidence suggests that approximately 20% of patients with post‐stroke epilepsy develop pharmaco‐resistance to ASM therapy, the risk of pharmaco‐resistance being associated with several factors, including younger age at stroke onset, history of intracranial hemorrhage, status epilepticus at initial presentation of epilepsy, and a shorter latency from stroke to epilepsy onset.^39^, ^40^ There are five previous or ongoing studies aiming to prevent post‐stroke epilepsy, all of them using ASMs (Table 2).^28^, ^41^, ^42^, ^43^, ^44^

1. A previous study of valproic acid in patients with non‐traumatic, non‐aneurysmatic spontaneous intracerebral hemorrhage used the same treatment duration as our study (30 days), but initiated treatment within 4 hours of the index event, and evaluated seizure rate and neurological function (assessed using the NIHSS) over 1 year (Table 2).^41^ Although the study found no difference between valproic acid and placebo in the risk of post‐stroke seizures (risk ratio, 0.88) or death (risk ratio, 1.20), it demonstrated that early treatment with valproic acid reduced the occurrence of early seizures and may have conferred some neuroprotective effect.^27^, ^41^
2. The Early Treatment with Levetiracetam After Stroke for the prevention of late seizures (ETLAS) study was a multicenter, randomized, placebo‐controlled, double‐blind study in which stroke patients with a cortical syndrome and a modified Rankin score ≥3 or NIHSS ≥6 were treated with levetiracetam 1500 mg/d or placebo for 12 weeks following stroke and followed up over 1 year (Table 2).^28^ Treatment was initiated between 48 hours and 7 days following the index event, and the primary endpoint was the occurrence of a first late epileptic seizure, defined as an unprovoked epileptic seizure >1 week after stroke.^28^ Problems with recruitment meant that between August 2005 and December 2006 only 16 patients (levetiracetam, n = 9; placebo; n = 7) were included in the study, and only one patient (placebo group) developed post‐stroke epilepsy.^28^ Recruitment was hampered because most patients met exclusion criteria or had comorbidities that precluded inclusion for practical reasons.^28^ Other problems encountered in the study were difficulties in recognizing and assessing the occurrence of epileptic seizures, the use of benzodiazepines and ASMs prescribed mostly by other treating physicians as comedications (which may have had anticonvulsive effects), and the evaluation of side‐effects occurring during the administration of study medication, which may have been related to the stroke, or to comedication initiated prior to or at the onset of stroke, rather than to the study medication.^28^ Due to difficulties such as these, the authors concluded that it is not feasible to conduct a prophylactic study assessing the antiepileptogenic efficacy of a short ASM treatment period to prevent post‐stroke epilepsy.^28^
3. The Early GABA‐ergic Activation Study In Stroke (EGASIS) was a randomized, double‐blind, placebo‐controlled study in which patients were treated with diazepam or placebo within 3–12 hours of acute stroke, with treatment continued for 3 days (Table 2).^42^ A substudy of EGASIS assessed the effectiveness of diazepam in primary seizure prevention in 784 patients following acute ischemic or hemorrhagic stroke over a follow‐up duration of up to 3 months.^43^ Although there was no statistically significant difference in seizure occurrence between patients treated with diazepam versus placebo (for all patients and the subgroups with ischemic or hemorrhagic stroke), a subanalysis of patients with cortical infarct in the anterior circulation demonstrated a statistical difference in favor of diazepam treatment (risk ratio, 0.21).^27^, ^43^ There was no significant difference in mortality between the diazepam and placebo groups after 2 weeks (risk ratio, 0.84) or 3 months (risk ratio, 0.95).^27^, ^43^
4. A Phase II, randomized, double‐blind, placebo‐controlled study has been designed to evaluate the efficacy and safety of perampanel as an antiepileptogenic treatment in up to 328 patients with cortical ischemic stroke or lobar hemorrhage (Table 2).^44^ Patients are randomized to receive perampanel (titrated to 6 mg/d over 4 weeks) or matching placebo, initiated within 7 days of stroke onset and continued for 12 weeks after titration.^44^ The primary outcome is the proportion of patients free of late post‐stroke seizures (defined as occurring >7 days after stroke onset) over 12 months.^44^ Exploratory outcomes will include evaluation of potential correlations between cerebral and plasma glutamate concentration and stroke and seizure outcomes, using 7T magnetic resonance imaging to quantify cerebral glutamate by magnetic resonance spectroscopy and glutamate chemical exchange saturation transfer imaging.^44^
5. A further study has been designed to assess whether early primary prophylactic treatment with low‐dose levetiracetam or perampanel, in comparison with placebo, can prevent the development of post‐stroke epilepsy in up to 180 patients with moderate to severe middle cerebral artery infarct (NIHSS >8), confirmed by brain magnetic resonance imaging studies (excluding lacunar infarct) during hospital admission (Table 2). The primary endpoint is the occurrence of epileptic seizures following acute stage of stroke, defined as occurring ≥14 days after the index event. The study is currently recruiting patients and no further information on its design is currently available.

**TABLE 2:** Comparative summary of Study BIA‐2093‐213 and other antiepileptogenic studies of ASMs following stroke.

ASM(s) (trial name)	Clinical trial identifier	Sponsor/center	Study design	Primary endpoint (timepoint)	Timing of treatment initiation after stroke	Treatment duration	Duration of follow‐up	Total number of patients planned (total number recruited [if completed])	Status and key results (if completed)
Eslicarbazepine acetate (Study BIA‐2093‐213)	EudraCT 2018‐002747	BIAL – Portela & Cª, S.A.	Phase II, multicenter, placebo‐controlled RCT	Failure rate (6 mo)	Within 120 h	30 d	Up to 18 mo	200 (125)	Ongoing
Valproic acid^41^	ClinicalTrials.gov NCT01115959	Faculty of Medicine, Tel Aviv University, Tel Aviv, Israel	Phase IV, single‐center, placebo‐controlled RCT	Seizure rate (12 mo)	At 2 (+2) h from intracerebral hemorrhage^a^	1 mo	12 mo	84 (72)	Completed No difference between valproic acid and placebo in risk of post‐stroke seizures (risk ratio, 0.88) or death (risk ratio, 1.20)
Levetiracetam^28^ (ETLAS)	–	University Hospital Maastricht, Maastricht, The Netherlands	Multicenter, placebo‐controlled RCT	US rate (12 mo)	48 h to 7 d	12 wk (maintenance)	12 mo	400 (16)	Completed Too few participants recruited to draw conclusions
Diazepam^42^, ^43^ (EGASIS)	–	University Hospital Maastricht, Maastricht, The Netherlands	Multicenter, placebo‐controlled RCT	Seizure occurrence^b^ (3 mo)	≤12 h^c^	3 d	3 mo	(784)^d^	Completed No statistically significant difference in seizure occurrence between patients treated with diazepam versus placebo (risk ratio, 0.47) No statistically significant difference in mortality between patients treated with diazepam versus placebo at 2 wk (risk ratio, 0.84) and 3 mo (risk ratio, 0.95) Primary prophylaxis with diazepam was associated with a reduced risk of post‐stroke seizures in patients with anterior circulation cortical infarcts (risk ratio, 0.21)
Perampanel^44^ (PEPSTEP)	ClinTrial Refer ACTRN12618001984280	IIS supported by Eisai	Phase II, multicenter, placebo‐controlled RCT	Seizure freedom (12 mo)	Within 7 d	16 wk (4 wk titration; 12 wk maintenance)	12 mo	Up to 328	Open
Perampanel Levetiracetam	ClinicalTrials.gov NCT04858841	National Cheng Kung University, Taiwan	Randomized, double‐blind case–control study	US rate (not available)	Not available	Not available	Not available	180	Recruiting

Abbreviations: ASM, antiseizure medication; h, hours; IIS, investigator‐initiated study; RCT, randomized controlled trial; US, unprovoked seizure.

^a^ Time to start of dosing after randomization was 14 ± 4 h in the valproic acid group and 16 ± 5 h in the placebo group.

^b^ Occurrence of seizures was registered prospectively as one of the prespecified secondary outcomes (primary endpoint was independence [modified Rankin score < 3] at 3 mo).

^c^ Preferably within 3 h but at most within 12 h.

^d^ This was an exploratory antiepileptogenesis substudy of the EGASIS Trial, for which a planned sample size was not prespecified; 784 patients were included in the substudy (diazepam, n = 389; placebo, n = 395).

What are the key requirements for a potential antiepileptogenic drug?

Firstly, preclinical findings must provide a strong rationale for investigating the potential antiepileptogenic effects of an ASM in the clinical setting. In animal models of acquired epilepsy, several medications in clinical use for diverse indications have been shown to have an antiepileptogenic effect, including medications with excellent side‐effect profiles.^45^ ESL is one of those compounds. Following oral administration, ESL is stereo‐selectively metabolized to its active metabolite, eslicarbazepine, which accounts for approximately 94% of plasma drug exposure.^46^ Although the precise mechanism of action of eslicarbazepine is unclear, it is thought to act primarily by enhancing the slow inactivation of voltage‐gated sodium channels, and its affinity for these channels in the inactivated and resting states is thought to indicate an enhanced selectivity for inhibiting rapidly firing active “epileptic” neurons.^33^, ^46^, ^47^ In addition, eslicarbazepine has been shown to be capable of potently inhibiting neuronal T‐type calcium channels; in particular, by inhibiting high‐ and low‐affinity hCaV3.2 inward currents.^32^, ^33^ Experimental models have demonstrated that Ca_v_3.2 channels play a key role in the development of chronic epilepsy,^48^, ^49^ and eslicarbazepine has shown promising antiepileptogenic effects in preclinical models.^32^, ^33^ In an experimental pilocarpine model of chronic epilepsy, EEG monitoring revealed that transitory ESL treatment within the epileptogenic period (150 mg/kg for 6 weeks) resulted in a significant decrease in both the frequency and duration of epileptiform discharges at the chronic stage (i.e., 8 weeks after the end of the treatment).^32^ Additionally, in these pilocarpine‐injected mice, ESL treatment caused a significant decrease in mossy fiber sprouting into the inner molecular layer, an attenuated neuronal loss, and a significant reduction in coordination impairment.^32^ These preclinical data indicate that transitory ESL treatment may attenuate the functional and morphological sequelae of status epilepticus and thus may have a possible antiepileptogenic effect.^33^ The antiepileptogenic properties of ESL might be explained by its inhibition of neuronal T‐type calcium channel Ca_v_3.2 currents, since these play a major role in epileptogenesis.^48^, ^49^

Secondly, an antiepileptogenic drug taken prophylactically must be well tolerated, with minimal side‐effects and few interactions with other medications. An oral route of administration and once‐daily dose regimen are also important factors, since the average age of the stroke population is relatively high and patients are more likely to be burdened with polypharmacy, particularly with medications that are initiated for stroke risk reduction (statins, antihypertensives, anticoagulants, etc). Moreover, due to brain injury resulting from stroke, patients are at risk of alterations in cognition, with up to 75% experiencing executive dysfunction^50^ and 21%–74% experiencing cognitive impairment.^51^, ^52^, ^53^, ^54^ Once‐daily administration would help with compliance, and, ideally, there should be no need for up‐titration, given the requirement for achieving effective doses quickly after the epileptogenic event. In view of the complexity of an antiepileptogenic study, repurposing of an existing compound in clinical use for either treatment of epilepsy or any other condition appears to be the only realistic option. ESL is a weak inducer of cytochrome P450 3A4 and uridine 50‐diphospho‐glucuronosyltransferase,^30^ and has a lower potential for drug–drug interactions than other ASMs in its class, such as carbamazepine and oxcarbazepine.^55^ It is administered orally as once‐daily tablets, which can be taken with or without food.^30^ Importantly, in patients with swallowing difficulties, the tablets may be crushed and mixed with water or soft foods immediately prior to use.^30^ ESL has a well‐defined and relatively favorable safety profile,^30^, ^46^ with no specific safety issues identified in elderly patients, in comparison with non‐elderly patients, in clinical trials and following long‐term post‐marketing surveillance.^56^, ^57^

These preclinical findings and considerations regarding the key requirements of an antiepileptogenic drug provide a strong rationale for investigating the potential antiepileptogenic effects of ESL in the clinical setting. However, there are key obstacles when designing such an antiepileptogenic study.

Firstly, a study with an unselected population of stroke patients would require a large sample size. This makes a study with a potentially antiepileptogenic compound difficult to operationalize and costly, as post‐stroke epilepsy does not develop in more than 15% of patients.^12^ Moreover, the benefit/risk ratio of such a study is questionable, since all ASMs have side‐effects and only a low proportion of patients will develop post‐stroke epilepsy; for example, in patients with a SeLECT score of ≤4, the risk of developing post‐stroke epilepsy after 1 and 5 years has been reported to be 6% and 11%, respectively.^16^ We used modified criteria of the SeLECT score^16^ and CAVE score^37^ to enrich the study population and so reduce the number of participants to be included in this randomized controlled study.

Secondly, an operational challenge when conducting an antiepileptogenic study is the question of when to initiate ASM therapy. Although it might be considered rational to initiate treatment as soon as possible after the stroke event, there are practical considerations regarding the patient reaching a medical facility as soon as possible to receive acute treatment for the stroke event, and the logistics of subsequently obtaining informed consent for study participation prior to initiation of prophylactic ASM therapy. Antiepileptogenic studies have historically adopted a range of time frames for ASM initiation, ranging from ≤12 hours to up to 7 days following the stroke event (Table 2).^28^, ^41^, ^42^, ^43^, ^44^ We made the pragmatic decision to allow initiation of ESL treatment up to 5 days (120 hours) after the stroke event, as it was felt that this was short enough to ensure early commencement of prophylaxis but long enough to avoid hampering recruitment for the logistical reasons outlined above.

Thirdly, a study with a potential antiepileptogenic and disease‐modifying compound would require a long follow‐up period. Epilepsy can manifest more than 5 years after the epileptogenic stroke,^58^ but over 80% of post‐stroke epilepsy presents within 24 months, and the majority within the first 6 months after stroke.^16^, ^59^, ^60^, ^61^ The antiepileptogenic effects of any compound can only be assessed once treatment with this compound has stopped. In this context, it was pragmatically decided to give early prophylactic therapy with ESL for 30 days in patients at high risk of developing post‐stroke seizures and follow‐up with these patients for another 17 months without any ASM. Convincing data supporting this therapy duration are lacking.

In summary, the design of Study BIA‐2093‐213 differs from other previous and ongoing studies of ASMs in the post‐stroke antiepileptogenic setting, in terms of time frame for treatment initiation, length of treatment, duration of follow‐up, recruitment duration, and number of patients required to allow robust statistical analysis (Table 2). As outlined above, Study BIA‐2093‐213 aimed to address some of the known challenges associated with conducting an antiepileptogenesis trial, such as using modified criteria of the SeLECT and CAVE scores to reduce the number of patients required. A pragmatic approach to treatment initiation was chosen, whereby treatment is initiated within 5 days (120 hours) of the index event, earlier than some other trials,^28^, ^44^ but not so early as to be unfeasible from a practical perspective. Despite these considerations, we encountered challenges that extended the recruitment period significantly (from 13 to 30 months) and led to early termination of recruitment. A particular challenge was to conduct an epilepsy study in the stroke unit setting since this necessitated a multidisciplinary approach to identify eligible patients and commence treatment in the necessary window of time. Although slow, recruitment was relatively constant over a 30‐month period, even though this encompassed several waves of the COVID‐19 pandemic. However, it is likely that the pandemic had a deleterious impact on recruitment; for example, by reducing the ability and willingness of patients to seek, access, and receive medical treatment; disrupting laboratory/diagnostic testing, timely data collection, and compliance with protocol‐defined procedures; reducing the availability of study personnel; and hindering the ability of multidisciplinary teams to operate efficiently.^62^, ^63^, ^64^, ^65^ Despite these difficulties, 125 patients have been randomized into the study and will be followed up as planned over an 18‐month period.

Given the current lack of prophylactic therapies for individuals at risk of epileptogenesis, and the lack of evidence to support the use of ASMs for the primary or secondary prevention of seizures following stroke,^2^, ^28^, ^29^, ^66^ it is still of high interest to continue to invest in this topic.

## CONFLICT OF INTEREST STATEMENT

MJK reports personal fees as a speaker or consultant from Arvelle, BIAL, Eisai, GW Pharmaceuticals, Novartis, and UCB Pharma. ET reports personal fees from EVER Pharma, Marinus, Arvelle, Angelini, Argenx, Medtronic, Bial‐Portela & Cª, NewBridge, GL Pharma, GlaxoSmithKline, Boehringer Ingelheim, LivaNova, Eisai, UCB, Biogen, Sanofi, Jazz Pharmaceuticals, and Actavis. His institution received grants from Biogen, UCB Pharma, Eisai, Red Bull, Merck, Bayer, the European Union, FWF Osterreichischer Fond zur Wissenschaftsforderung, Bundesministerium für Wissenschaft und Forschung, and Jubiläumsfond der Österreichischen Nationalbank. YHM has no conflicts of interest to disclose. CB reports personal fees as a speaker or consultant from Angelini, BIAL, Medtronic, and Eisai. SK reports personal fees as a speaker or consultant from Angelini, Bial, Desitin, Eisai, Merck Serono, Precisis, UCB Pharma, and Zogenix. GLG reports personal fees from Angelini, Neuraxpharm, and Idorsia, and institutional support from Roche and Penta. JMS reports personal fees as a consultant/advisor from Arvelle Therapeutics, Angelini, BIAL, Eisai, Jazz Pharmaceuticals, Sanofi, UCB Pharma; and speaker honoraria from Arvelle Therapeutics, Angelini, BIAL, Eisai, Sanofi, UCB Pharma. JZ reports speaker honoraria for unbranded educations from Eisai and UCB, and as an employee of Sahlgrenska University (no personal compensation) being an investigator/subinvestigator in clinical trials sponsored by UCB, GW Pharma, Bial, and SK Life Science. LMM is an employee of Bial – Portela & Cª, S.A. AP was an employee of Bial – Portela & Cª, S.A. at the time this study was implemented but is currently employed at BlueClinical, Portugal. JM is an employee of Bial – Portela & Cª, S.A. PS is an employee of Bial – Portela & Cª, S.A. We confirm that we have read the Journal's position on issues involved in ethical publication and affirm that this report is consistent with those guidelines.
